# Copper(II) phosphate as a promising catalyst for the degradation of ciprofloxacin via photo-assisted Fenton-like process

**DOI:** 10.1038/s41598-024-57542-9

**Published:** 2024-03-25

**Authors:** Mateusz Rozmyślak, Adrian Walkowiak, Marcin Frankowski, Lukasz Wolski

**Affiliations:** Faculty of Chemistry, Adam Mickiewicz University, Poznań, ul. Uniwersytetu Poznańskiego 8, 61-614 Poznań, Poland

**Keywords:** Advanced oxidation processes, Photo-assisted Fenton-like process, Metal phosphate, Antibiotic degradation, Water treatment, Environmental chemistry, Catalysis

## Abstract

This work aims to unravel the potential of copper(II) phosphate as a new promising heterogenous catalyst for the degradation of ciprofloxacin (CIP) in the presence of H_2_O_2_ and/or visible light (λ > 400 nm). For this purpose, copper(II) phosphate was prepared by a facile precipitation method and fully characterized. Of our particular interest was the elucidation of the kinetics of CIP degradation on the surface of this heterogeneous catalyst, identification of the main reactive oxygen species responsible for the oxidative degradation of CIP, and the evaluation of the degradation pathways of this model antibiotic pollutant. It was found that the degradation of the antibiotic proceeded according to the pseudo-first-order kinetics. Copper(II) phosphate exhibited ca. 7 times higher CIP degradation rate in a Fenton-like process than commercial CuO (0.00155 vs. 0.00023 min^−1^, respectively). Furthermore, the activity of this metal phosphate could be significantly improved upon exposure of the reaction medium to visible light (reaction rate = 0.00445 min^−1^). In a photo-assisted Fenton-like process, copper(II) phosphate exhibited the highest activity in CIP degradation from among all reference samples used in this study, including CuO, Fe_2_O_3_, CeO_2_ and other metal phosphates. The main active species responsible for the degradation of CIP were hydroxyl radicals.

## Introduction

Pharmaceutical products are essential for human and livestock health. In particular, around 95% of the drugs currently used in modern medicine undergo only partial metabolic breakdown within the organisms and are subsequently released into the environment due to the low efficiency of their removal through conventional water purification techniques used in wastewater treatment plants^1^. Very recent reports have underlined that more than 100 distinct pharmaceutical compounds can be detected nowadays in surface and wastewater sources^2^. Although the immediate amounts entering aquatic ecosystems appear to be relatively low, their cumulative long-term impact has the potential to pose significant risks to these ecosystems^1^. Furthermore, pharmaceutical residues in waters can induce resistance to antibiotics in some strains of bacteria, thereby presenting a serious threat to public health in the future^3^. Among the various pharmaceuticals, ciprofloxacin is of particular significance in the medical field, as it plays a crucial role in the treatment of bacterial infections caused by both gram-positive and gram-negative bacteria^4^. Remarkably, ciprofloxacin has been detected in varying concentrations within different environmental compartments. For example, it has been found in amounts exceeding 150 µg/L in hospital wastewater and up to 50 mg/L in pharmaceutical effluent^5^.

Advanced Oxidation Processes (AOPs) are one of the most promising and environmentally benign methods for the removal of antibiotics from water. The term “advanced oxidation processes” encompasses a range of different types of oxidation processes, the main purpose of which is to produce highly oxidizing reactive oxygen species (ROS), such as hydroxyl radicals (HO^·^). Examples of advanced oxidation processes include Fenton and Fenton-like reactions employing H_2_O_2_ as a primary oxidant to be catalytically activated toward ROS formation^6^. The radicals produced as a result of the catalytic activation of H_2_O_2_ exhibit a high oxidizing potential and are able to mineralize complex organic pollutants, leading to their decomposition into simple inorganic compounds that no longer pose a threat to human health and life^7^. However, the homogeneous Fenton process, in which Fe^2+^ ions are used as a catalyst, has several significant drawbacks that limit its wide application. Among the most significant are the decrease in system reactivity at a pH greater than 3.5 due to the precipitation of iron cations in the form of Fe(OH)_x_^8^, and the difficulty in the separation of the homogeneous catalyst from the post-reaction mixtures after water purification^8^. Due to the drawbacks described above associated with the applicability of the homogeneous Fenton process for the removal of organic pollutants from water, considerable attention has been devoted in recent years to the search for new heterogeneous catalysts that would be able to effectively remove organic contaminants over a wider range of pH values (pH close to neutral) and that, upon completion of the reaction, could be easily separated from the purified water. To date, most fundamental studies in this field have been carried out using metal oxide-based catalysts such as Fe_2_O_3_, CuO, or CeO_2_, which are known for their unique ability to activate H_2_O_2_ towards ROS via a Fenton-like mechanism and enable efficient degradation of various organic pollutants^9,10^. Current research in this field is focused on improving the activity of these heterogeneous catalysts in ROS generation and degradation of organic pollutants. One way to achieve this goal is to design novel catalysts that contain phosphate ions. To date, it has been documented that incorporation of phosphate species into the structure of niobia not only increased the sorption capacity of this metal oxide toward methylene blue (MB), but also allowed a much higher efficiency of H_2_O_2_ activation toward the formation of singlet oxygen. The latter was identified as the main ROS responsible for strongly enhanced reactivity of phosphate-doped niobia in MB discoloration^11^. Furthermore, Xiaoya et al.^12^ have revealed that the presence of a phosphate modifier in the PO_4_/H-ZSM-5/BiOCl composite resulted in a more efficient adsorption of carbamazepine (CBZ). Additionally, the phosphate modifier enabled the formation of BiPO_4_ on the surface of BiOCl. The combined effects of the enlarged surface area, the enhanced sorption capacity, and the formation of a new BiPO_4_ phase in the composite catalyst resulted in a more efficient separation of photo-generated charge carriers, and thus, its highly improved activity in the photocatalytic degradation of CBZ. Concerning other metal phosphates, recent studies by Fijołek and Wolski^13^ have revealed that the catalysts containing CePO_4_ are very promising nanomaterials for the degradation of organic pollutants through ozonation. The authors have found that CePO_4_ is not only more active in the degradation of benzoic acid than CeO_2,_ but also enables more efficient mineralization of the target pollutant. A similar phenomenon has also been reported for CePO_4_-containing catalysts during catalytic activation of H_2_O_2_^14^. However, to the best of our knowledge, very little attention has been paid to copper(II) phosphate-based catalysts and their use in AOPs. This metal phosphate exhibits several desirable properties and has been successfully applied in methane oxidation^15^, selective oxidation of benzylic alcohols^16^ and photocatalytic degradation of methylene blue^17^. However, there is no information on the use of this nanomaterial in Fenton-like and photo-Fenton-like processes aiming at the degradation of antibiotic pollutants. This study aims to fill the above-mentioned gap in the fundamental knowledge and provides a deep insight into the activity of copper(II) phosphate in the degradation of CIP via the Fenton-like and photo-assisted Fenton-like processes. The particular objectives of our work were the following: (i) evaluation of the kinetics of CIP degradation on the surface of this heterogenous catalyst, (ii) identification of the main ROS responsible for oxidative degradation of CIP, and (iii) analysis of the degradation pathways of this model antibiotic pollutant. To clearly underline the potential of the copper(II) phosphate as a novel catalyst dedicated to AOPs, the activity of this nanomaterial in the degradation of CIP was compared to those observed for other metal oxide-based catalysts, which are known as efficient catalysts dedicated to the degradation of organic pollutants through Fenton-like and photo-assisted Fenton-like processes, namely CuO, Fe_2_O_3_, and CeO_2_. Of particular interest was also the analysis of catalyst stability and the description of the effect of catalyst loading and H_2_O_2_ dosage on the efficiency of CIP degradation.

## Methods

### Materials

Copper(II) nitrate trihydrate (Cu(NO_3_)_2_·3H_2_O, Sigma-Aldrich, 99–104%), ammonium phosphate dibasic ((NH_4_)_2_HPO_4_, Sigma-Aldrich, ACS Reagent, ≥ 98%), ciprofloxacin (CIP, Sigma-Aldrich, ≥ 98%, HPLC grade), commercial copper(II) oxide (CuO, Sigma-Aldrich, nanopowder, < 50 nm particle size (TEM)), hydrogen peroxide (H_2_O_2_, Sigma-Aldrich, 30%), cerium(III) nitrate hexahydrate (Ce(NO_3_)_3_·6 H_2_O, Sigma-Aldrich, ACS Reagent, 99.99%), nitric acid (HNO_3_, 65%, Chempur, ACS Reagent), sodium hydroxide (NaOH, POCH, ACS Reagent), isopropyl alcohol (STANLAB, analytical grade), were all used without any further purification. Deionized (DI) water was used throughout the experiments.

### Synthesis of catalysts

Copper(II) phosphate (Cu_3_(PO_4_)_2_) was synthesized using a facile precipitation method. For this purpose, 2.4160 g (0.01 mol) of Cu(NO_3_)_2_ and 2.6412 g (0.02 mol) of (NH_4_)_2_HPO_4_ were dissolved in 100 mL portions of deionized water. Then, the solution containing ammonium phosphate dibasic was stirred into an aqueous solution of copper(II) nitrate. The precipitation reaction occurred immediately after mixing the solutions and resulted in the formation of a light-blue solid. After 1 h of intensive agitation, the precipitate was separated by filtration, washed with 500 mL of DI water and dried at 80 °C for 24 h.

The synthesis of all reference materials, including CeO_2_, CePO_4_, Fe_2_O_3_ and Fe_2_O_3_ doped with phosphate ions (P:Fe_2_O_3_) is described in the extended experimental section (see Supporting Information).

### Characterization of catalysts

Nitrogen adsorption–desorption isotherms were recorded using a Quantachrome instrument at − 196 °C. Samples were degassed at 120 °C for 12 h before measurement. The specific surface area value was calculated using the Brunauer–Emmett–Teller (BET) method. The pore size distribution was determined using the BJH (Barrett-Joyner-Halenda) method.

The XRD patterns were recorded on a D8 Advance diffractometer (Bruker) using CuKα radiation (λ = 0.154 nm), with a step size of 0.02° in the 2θ range of 10–80°.

The morphology of the synthesized catalysts was investigated using a field-emission scanning electron microscope (FESEM) Quanta 250 FEG, FEI operating at an accelerating voltage of 10 kV. Energy dispersive X-ray analysis (EDX) and EDX elemental mapping were performed using the EDX analyzer and beam accelerating voltage of 30 kV. All measurements were conducted on a carbon adhesive conductive tape, without metallization.

Diffuse reflectance (DR) UV–vis spectra were recorded on a Varian Cary 300 Scan spectrophotometer equipped with a diffuse reflectance accessory. Spectra were acquired at room temperature in the spectral range of 200–800 nm. Spectralon was used as a reference material.

Infrared spectroscopy (FT-IR) measurements were made using a Bruker Vertex 70 spectrometer. Before measurements, all samples were mixed with KBr (5 mg of the sample and 200 mg of KBr), homogenized using agate mortar, and 100 mg of the resulting mixture was pressed into a self-supporting pellet. FT-IR spectra were recorded in the range of 4000–400 cm^−1^.

X-ray photoelectron spectroscopy (XPS) was performed using an ultra-high vacuum photoelectron spectrometer based on a Phoibos150 NAP analyzer (Specs, Germany). The analysis chamber was operated under vacuum at a pressure close to 5 × 10^−9^ mbar and the sample was irradiated with a monochromatic AlKα (1486.6 eV) radiation. Any charging that might occur during the measurements was accounted for by rigidly shifting the entire spectrum by a distance needed to set the binding energy of the C1s, assigned to adventitious carbon, to the assumed value of 284.8 eV.

Measurements of the zeta potential as a function of the pH of aqueous dispersions of the studied samples were performed on a Zetasizer Nano ZS instrument (Malvern). The zeta potential was estimated from electrophoretic mobility using the Henry equation: U_E_ = 2εζF(ka)/3η, where U_E_ is the electrophoretic mobility, ζ the zeta potential, ε the dielectric constant, F(ka) Henry’s function (set for 1.5 as in the Smoluchowski’s approximation), and η the viscosity. The pH value was adjusted with 0.1 mol/L of HCl or NaOH solutions.

### Catalytic activity test

All catalytic tests were performed using an EasyMax 102 Advanced Thermostat system (Mettler Toledo) at room temperature. The highest concentration of CIP used in the catalytic tests was 15 mg/L. In this concentration range, a linear correlation was observed between absorbance (λ_max_ = 271 nm) and CIP concentration (see Fig. S1A and B). In a typical reaction, 25 mg of a given catalyst was added to a glass reactor (total volume of 150 mL) containing 100 mL of aqueous ciprofloxacin solution (15 mg/L, native pH ~ 6.5). Then, to initiate the reaction, 50 µL of aqueous solution of hydrogen peroxide (30%) was added. The reactions were performed in a dark chamber to avoid photocatalytic degradation of CIP in Fenton-like processes. In the case of photocatalytic and photo-assisted Fenton-like processes, the reactor was irradiated from the top using a 200 W Hg-Xe lamp (Hamamatsu LC8 spot light) equipped with a UV cut-off filter (transmissive to light above 400 nm only) and a light guide (model: A10014-50-0110). In the upper part of the reactor (6 cm from the end of the light guide), the intensity of light was of 0.24 W/cm^2^ and it decreased to 0.08 W/cm^2^ at the lower part of the glass reactor (11 cm from the end of the light guide). CIP removal was monitored using UV–Vis spectroscopy (Varian, Cary 300). For this purpose, after a given reaction time, 4 mL of the mixture was withdrawn from the reactor and the catalyst was filtered off through a syringe filter 0.2 μm Whatman (hydrophobic, PTFE). No noticeable changes in CIP concentration were observed after the filtration process. As shown in Fig. S1C and D, the presence of a small amount of H_2_O_2_ in the reaction medium (from 10 to 100 μL of concentrated H_2_O_2_ per 100 mL of CIP solution) had a negligible influence on the absorbance of the CIP solution at 271 nm. Therefore, the removal of CIP in the presence of H_2_O_2_ could be correctly estimated on the basis of UV–vis measurements.

The CIP degradation products were identified with the use of an LC–MS/MS 8050 instrument (Shimadzu, Japan) in a positive ion mode. Samples were injected into the ESI source with a SIL 30AC autosampler (sampling speed 5 µL/s. for 1 µL injection volume) and 30/70 H_2_O/ACN (1% formic acid) mobile phase. The ESI conditions were as follows: nebulizing gas flow rate: 3 L/min, heating gas flow rate: 10 L/min, drying gas flow rate: 10 L/min, interface temperature: 300 °C, DL temperature: 250 °C, heat block temperature: 400 °C.

The concentration of total organic carbon (TOC) in the reaction mixtures was analyzed with the use of a Total Organic Carbon analyzer (TOC-L) (Shimadzu, Japan). The concentration of copper species leached from a catalyst during catalytic reactions was determined by Inductively Coupled Plasma—Optical Emission Spectrometry (ICP-OES). For this purpose, the catalyst was separated from the post-reaction mixture by filtration through a 0.2 μm Millipore filter (PTFE, hydrophobic) and the concentration of selected elements in the filtrate was quantified by ICP-OES spectrometer (Shimadzu, Japan).

## Results and discussion

### Physicochemical properties of the catalysts

**Figure 1:**
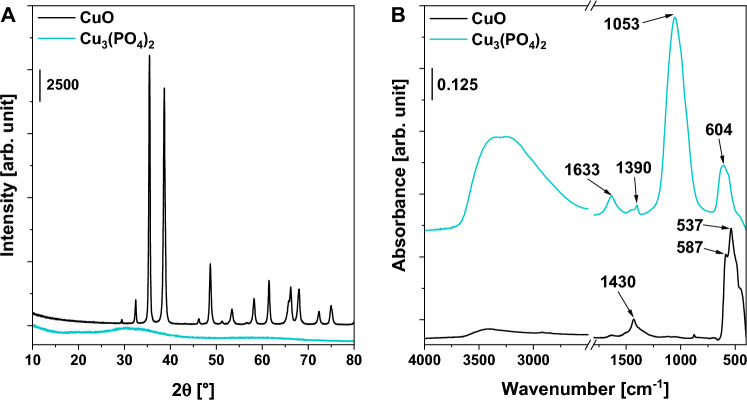
(**A**) XRD diffractograms and (**B**) FT-IR spectra of the catalysts.

Figure 1A shows the XRD pattern of the as-synthesized copper(II) phosphate and the commercial CuO that was used as a reference material. The latter material exhibits a well-defined crystalline structure typical of the tenorite phase (ICDD no. 04-007-0518, Fig. S2). The former sample was amorphous and no peaks characteristic of any specific crystalline phase were detected. Thus, the identification of chemical composition and structure of copper(II) phosphate was impossible solely on the basis of XRD measurements.

More detailed information on the structure of copper(II) phosphate was obtained from FT-IR studies. As shown in Fig. 1B, the IR spectrum of CuO reveals the presence of two absorption bands at ca. 537 cm^−1^ and 587 cm^−1^ which are characteristic of Cu–O stretching vibrations in the structure of CuO^18^, and one less intense band at ca. 1430 cm^−1^ which is associated with antisymmetric stretching vibration of –ONO_2_^19^. The latter band results more likely from the presence of some impurities originating from the metal source (copper(II) nitrate) used during the synthesis of the commercial sample. In the case of copper(II) phosphate, the most intense band was observed at 1053 cm^−1^ and was assigned to the asymmetric vibrations of the P–O stretching in PO_4_^3–^ groups^20^. The IR spectrum of this catalyst also revealed the presence of two additional bands at 604 cm^−1^ and 1633 cm^−1^ which are characteristic of O–P–O^21^ and P–OH^22^ vibrations, respectively. A broad absorption band was observed at ca. 3300–3500 cm^−1^ can be assigned to –OH species in PO_4_^3–^ groups and/or physiosorbed water molecules^23^. In the case of copper(II) phosphate, the IR band characteristic of –OH vibrations was much more intense than that observed for CuO. This phenomenon results, to some extent, from the robust affinity of phosphate species for hydrogen bonding with water molecules and their effective binding to the surface of copper(II) phosphate^24^.

**Figure 2:**
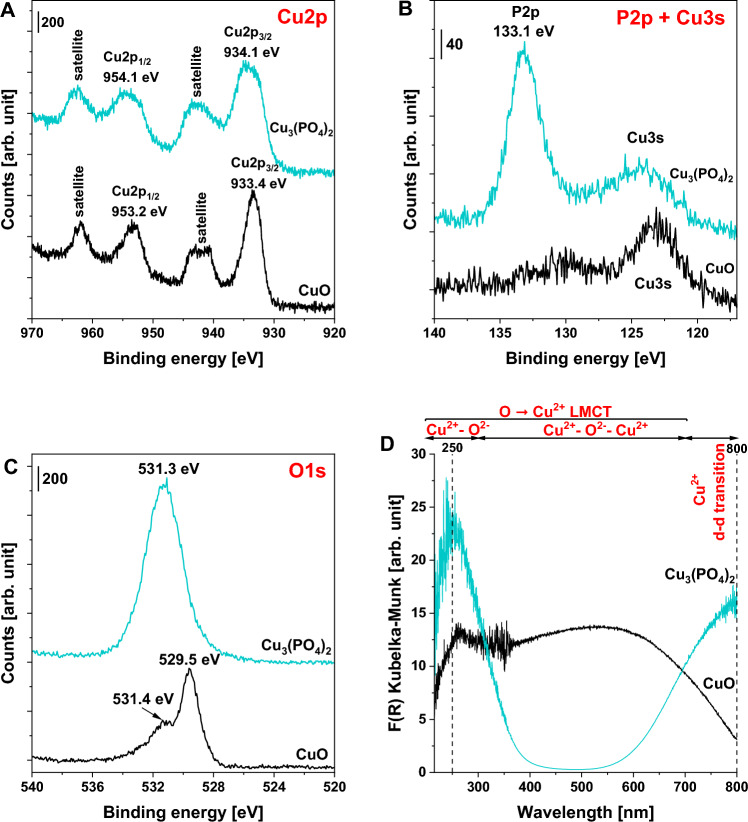
XPS spectra of CuO and Cu_3_(PO_4_)_2_ catalysts in different binding energy regions: (**A**) Cu 2*p*, **(B)** P 2*p* + Cu 3*s* and (**C**) O 1*s*. (**D**) DR UV–vis spectra of the catalysts.

The successful formation of copper(II) phosphate was also confirmed by XPS measurements. As shown in Fig. 2A, the XPS spectrum of CuO in the Cu 2*p* binding energy region revealed the presence of four spectral components. The peaks at 933.4 and 953.2 eV are characteristic of Cu^2+^ species in CuO (spectral components Cu 2*p*_3/2_ and Cu 2*p*_1/2_, respectively)^25^. The two remaining peaks are assigned to Cu^2+^ satellites. In the case of copper(II) phosphate, the Cu 2*p*_3/2_ and Cu 2*p*_1/2_ peaks were shifted toward noticeably higher binding energy values (934.1 and 954.1 eV, respectively) when compared to that of CuO, indicating a different chemical environment of Cu^2+^ species in this material. More pronounced differences were observed in the binding energy regions of P 2*p* and O 1*s*. As shown in Fig. 2B, the XPS spectrum of copper(II) phosphate shows one very intense peak located at 133.1 eV and a less intense peak at ca. 124 eV. According to the literature^26^, the former peak is associated with PO_4_^3–^ species in metal phosphates. The latter peak at the higher energy value (ca. 124 eV) is assigned to the Cu 3*s* region, which overlaps with that characteristic of P 2*p*. Regarding the XPS spectra in the O 1*s* region, two well-distinguished peaks characterized by a binding energy of 529.5 and 531.4 eV were observed for the commercial CuO (see Fig. 2C). According to the literature ^27^, they are assigned to the lattice oxygen in CuO, and surface oxygen (e.g., surface hydroxyl groups) and/or the oxygen in physiosorbed water molecules, respectively. In the case of copper(II) phosphate, one can observe only one broad and symmetric peak at ca. 531.3 eV, which is characteristic of oxygen species in PO_4_^3–^ ions^28^ (Fig. 2C). No noticeable spectral component at the binding energy typical of the lattice oxygen in CuO was found. On the basis of the above, one can clearly conclude that the amorphous material obtained during the synthesis can be undoubtedly assumed as amorphous copper(II) phosphate.

Significant differences were also noticed between commercial CuO and copper(II) phosphate in terms of their optical properties. As shown in Fig. 2D, the DR UV–vis spectrum of copper(II) phosphate shows two intense bands at ca. 250 and 800 nm. The former is attributed to the ligand-to-metal charge transfer (LMCT) from O^2–^ to Cu^2+^ species^29–33^, while the latter is typical of *d*−*d* transitions of Cu^2+^ in a distorted octahedral structure, in which the interactions between Cu^2+^ and polyhedral neighbours, such as phosphate groups, lead to a progressively distorted octahedral symmetry^29,31,34^. In the case of commercial CuO, one can observe only the former peak, typical of the Cu^2+^–O^2–^ LMCT transitions, at ca. 250 nm, and additionally a broad absorption band in the range of 300–700 nm, which is assigned to oligomeric Cu–O–Cu bonds in the structure of CuO^31^. The absence of the latter broad band in the DR UV–vis spectra of copper(II) phosphate indicates that no copper(II) oxide phase existed in this material. Thus, the DR UV–vis data further confirm the successful formation of pure copper(II) phosphate without any impurities resulting from the presence of CuO species.

**Figure 3:**
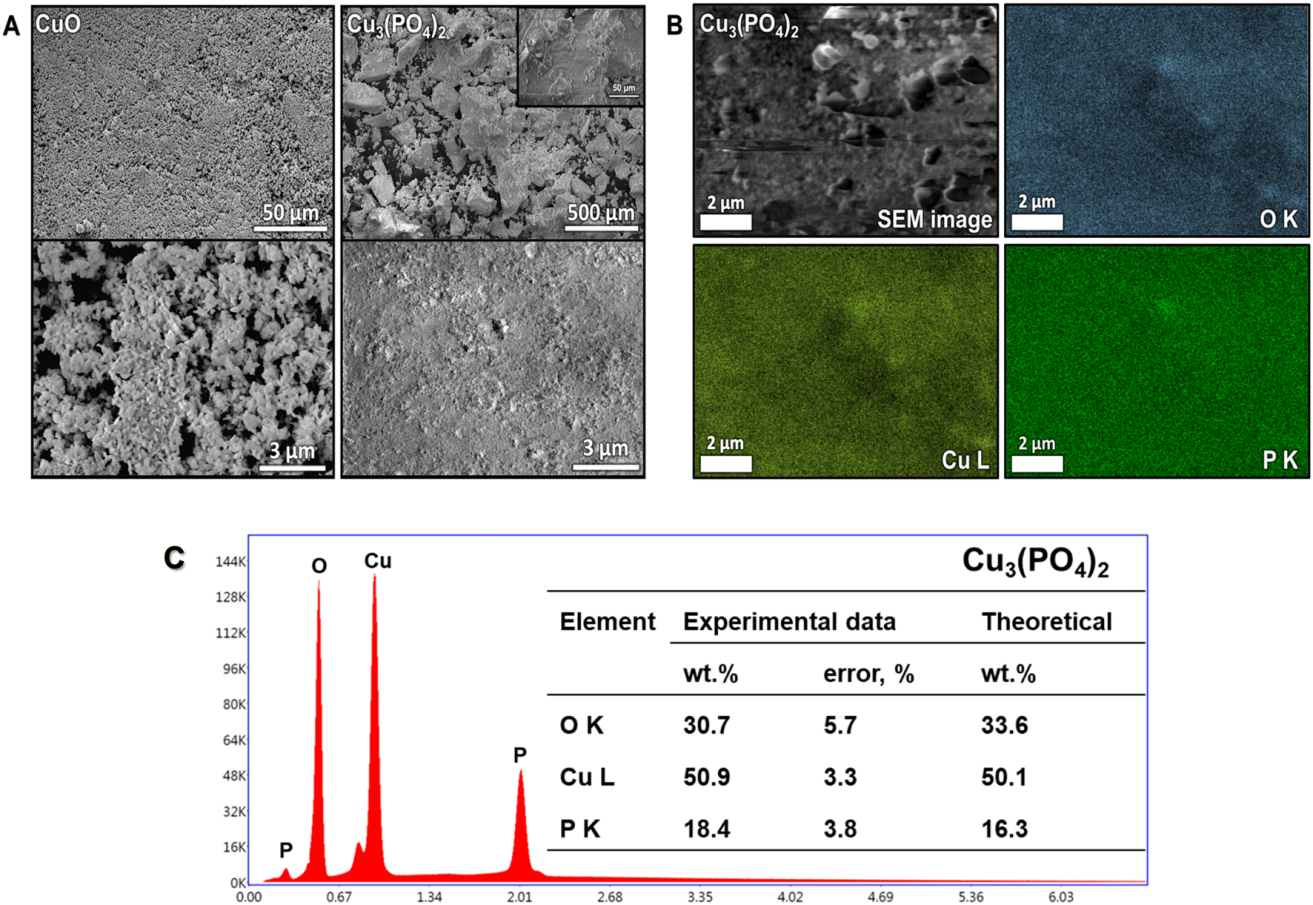
(**A**) SEM images of CuO and Cu_3_(PO_4_)_2_ catalysts. (**B**,**C**) Results of SEM–EDX mapping for Cu_3_(PO_4_)_2_.

In order to confirm the formation of copper(II) phosphate on a microscale, SEM measurements combined with EDX mapping were carried out. As shown in Fig. 3A, CuO consisted of spherical particles fused into larger aggregates to form a porous structure. In the case of copper(II) phosphate, particles of this material were much smaller and fused into irregular aggregates of different sizes, with sharp edges. Concerning the distribution of individual elements in Cu_3_(PO_4_)_2_, the results of EDX mapping shown in Fig. 3B revealed that Cu, O and P were homogeneously distributed on the surface of this catalyst. There were no regions in which only Cu or P species were detected. These observations are in agreement with the conclusions drawn on the basis of XRD, FT-IR, DR UV–vis and XPS studies, and further confirm that this material contained only the copper(II) phosphate phase and no noticeable amount of copper(II) oxide was found. The formation of pure Cu_3_(PO_4_)_2_ was also confirmed by the EDX results. As shown in Fig. 3C, the chemical composition established on the basis of the SEM–EDX measurements is in a good agreement with the theoretical composition of this metal phosphate.

**Figure 4:**
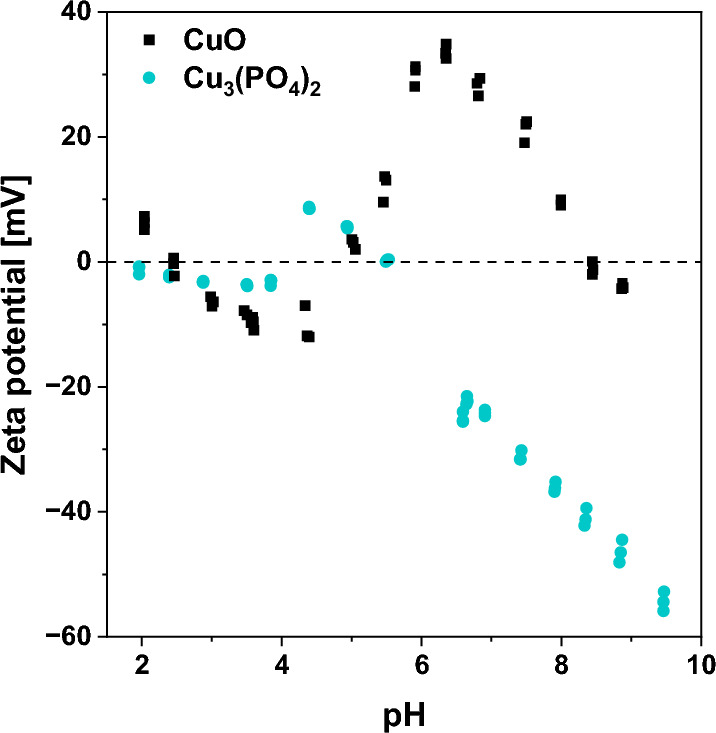
Zeta potential measurements of CuO and Cu_3_(PO_4_)_2_ catalysts.

To obtain more precise information on the stability and surface properties of the materials in the liquid phase, zeta potential measurements were performed. As shown in Fig. 4, copper(II) phosphate exhibited a totally different surface charge in aqueous media than commercial CuO. For example, the surface of CuO was positively charged at a pH above 6, while under the same conditions, copper(II) phosphate exhibited negative surface charge. The negative charge at pH close to neutral observed for copper(II) phosphate more likely resulted from the presence of phosphate ions in its structure. A similar surface charge has previously been reported for other metal phosphates, including YPO_4_^35^, and Nb_2_O_5_ doped with phosphate ions^11^. These observations further confirm completely different chemical composition and surface properties of the investigated materials.

### Catalytic activity

**Figure 5:**
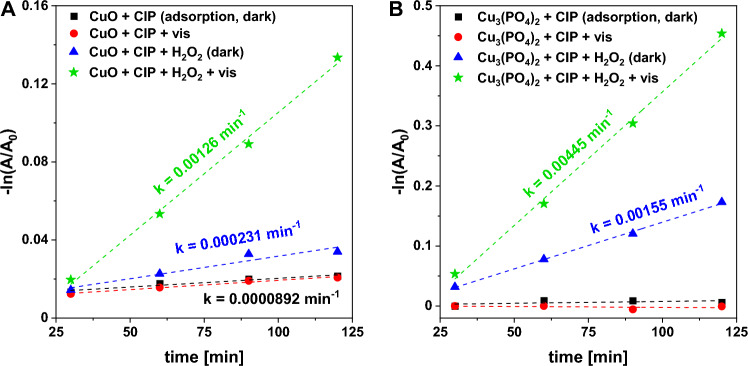
Pseudo-first-order plot for determination of apparent CIP degradation rate under different conditions, in the presence of (**A**) CuO and (**B**) Cu_3_(PO_4_)_2_ catalysts. *Reaction conditions:* catalyst (25 mg), H_2_O_2_ (50 μL, 30%), CIP (100 mL, 15 mg/L), room temperature, stirring rate (600 rpm), visible light (λ ≥ 400 nm), without pH adjustment (native pH ~ 6.5).

The catalytic activities of copper(II) phosphate and the reference CuO catalyst were tested in the degradation of ciprofloxacin as a model antibiotic pollutant. All reactions were carried out under conditions in which relatively low CIP removal (below 40%) was observed (Fig. S3) to enable a reliable analysis of the reaction kinetics. As shown in Fig. 5, both copper(II) phosphate and copper(II) oxide did not exhibit any noticeable photocatalytic activity under the applied reaction conditions. Interestingly, much higher activity of CuO and Cu_3_(PO_4_)_2_ was observed for the reaction with the use of H_2_O_2_ as an oxidant (Fenton-like process). In both cases, CIP degradation in a Fenton-like reaction followed the pseudo-first order kinetics (Fig. 5). The CIP degradation rate observed for copper(II) phosphate was approximately seven times higher than that established for the commercial CuO (0.00155 vs. 0.00023 min^–1^, respectively; Fig. 5). A similar tendency was also observed in a photo-Fenton-like reaction in which the copper(II) phosphate was ca. 3.5 times more efficient in antibiotic degradation than commercial CuO (Fig. 5).

To verify the potential of copper(II) phosphate as a promising catalyst for CIP degradation through the photo-Fenton process, the antibiotic degradation rate observed for this material was compared with that established for the other metal oxide-based nanomaterials commonly used as Fenton-like catalysts, including Fe_2_O_3_, CeO_2_, as well as metal phosphates/metal oxides doped with phosphate ions (i.e. CePO_4_, P:Fe_2_O_3_). XRD patterns that confirm the structure of these reference materials are shown in Fig. S4, while the physicochemical properties of these catalysts are summarized in Table 1. Among all reference materials, the highest BET surface area was observed for P:Fe_2_O_3_ (239 m^2^/g). It was approximately five times greater than that observed for copper(II) phosphate (57 m^2^/g). CeO_2_ and CePO_4_ exhibited a BET surface area similar to that of copper(II) phosphate, while Fe_2_O_3_ and commercial CuO had the lowest surface area of 16 and 7 m^2^/g, respectively.

**Table 1:** Physicochemical properties of copper(II) phosphate and all reference samples used in this study.

Catalyst	Structure	BET surface area [m^2^/g]^a^	Average pore size [nm]^b^
CuO	Tenorite	7	5.1
Cu_3_(PO_4_)_2_	Amorphous	57	58.5
Fe_2_O_3_	Hematite	16	40.9
P:Fe_2_O_3_	Amorphous	239	3.3
CeO_2_	Cubic	56	5.9
CePO_4_	Hexagonal	74	20.0

^a^Nitrogen adsorption–desorption isotherms are shown in Fig. S5.

^b^Average pore size estimated from the adsorption branch using the BJH method.

**Figure 6:**
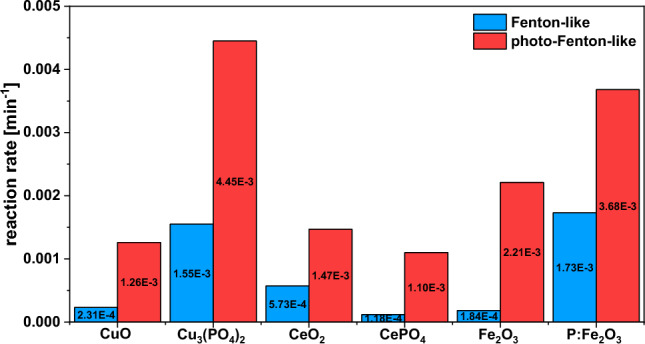
Comparison of CIP degradation rates in the presence of copper(II) phosphate catalyst and other nanomaterials known for their high reactivity in Fenton-like and photo-Fenton-like processes. *Reaction conditions:* catalyst (25 mg), H_2_O_2_ (50 μL, 30%), CIP (100 mL, 15 mg/L), room temperature, stirring rate (600 rpm), visible light (λ ≥ 400 nm), without pH adjustment (native pH ~ 6.5). The plots used for the determination of the reaction rate are shown in Fig. S6.

According to the results of catalytic tests, copper(II) phosphate was much more active in the degradation of CIP via Fenton-like process than the majority of the other metal oxides and phosphates used as reference materials (Fig. 6). For example, the CIP degradation rate observed in the Fenton-like process for Fe_2_O_3_ was approximately 13 times lower than that established for copper(II) phosphate. Further, cerium(III) phosphate (CePO_4_), which was found to be a very promising nanomaterial for the catalytic activation of H_2_O_2_^14^ and degradation of benzoic acid by ozonation^13^, and had almost the same surface area as copper(II) phosphate, exhibited much lower activity than Cu_3_(PO_4_)_2_. Only phosphate-doped Fe_2_O_3_ characterized by a much larger surface area than that observed for Cu_3_(PO_4_)_2_ (239 vs. 57 m^2^/g, respectively) was slightly more active in the CIP degradation in the dark (Fenton-like process; Fig. 6). However, a different phenomenon was observed in the photo-assisted Fenton-like process in which the latter sample significantly outperformed the former one. As shown in Fig. 6, copper(II) phosphate exhibited ca. 20% higher reaction rate in CIP degradation than Fe_2_O_3_ doped with phosphate ions (P:Fe_2_O_3_). It is important to emphasize that the CIP degradation rate observed for copper(II) phosphate in the photo-assisted Fenton-like process was ca. 3.5 times higher than that of commercial CuO and twice higher than that established for Fe_2_O_3_. This information provides grounds for the conclusion that copper(II) phosphate is a promising nanomaterial for the efficient degradation of CIP via the photo-assisted Fenton-like process. In terms of comparison with data from the previous literature, it was revealed that copper(II) phosphate allows a higher CIP degradation efficiency than various iron-molybdate-based zeolitic octahedral metal oxides^36^ or Fe_2_O_3_/MoO_3_ composites^36^ (see Table S1) applied at pH close to neutral (pH ~ 7). It also exhibits activity comparable to that of Corncob Biochar-Based Magnetic Iron–Copper Bimetallic Nanomaterial^37^ or HNO_3_ modified-biochar^38^. However, copper(II) phosphate is significantly less efficient than other iron-based nanomaterials applied under strongly acidic conditions (pH ~ 3), such as ferrocence supported on mesoporous silica SBA-15 (Fc/NH_2_/SBA-15)^39^ or C_3_N_4_/Fe_3_O_4_/MIL-100(Fe) ternary heterojunction^40^, which reached similar CIP degradation in a significantly shorter reaction time (see Table S1).

**Figure 7:**
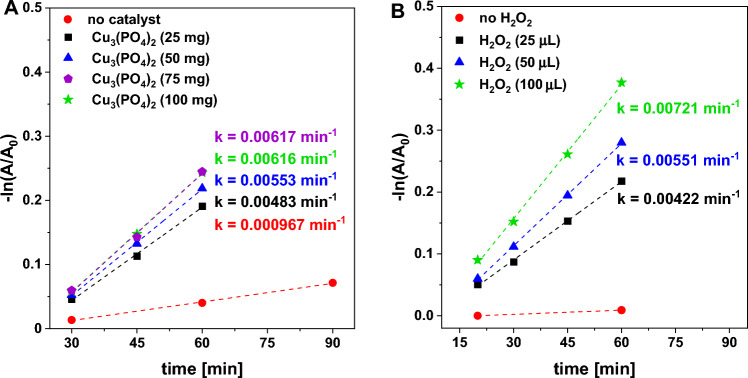
Pseudo-first-order plot for determination of the apparent CIP degradation rate in a photo-assisted Fenton-like process in the presence of (**A**) various loadings of the catalyst, and (**B**) with the use of different initial concentrations of H_2_O_2_. *Reaction conditions:* catalyst (Cu_3_(PO_4_)_2_, 25 mg, or other if indicated), H_2_O_2_ (50 μL, 30%, or other if indicated), CIP (100 mL, 15 mg/L), room temperature, stirring rate (600 rpm), visible light (λ ≥ 400 nm), without pH adjustment (native pH ~ 6.5).

More detailed studies aimed at the evaluation of the mechanism of the catalytic process and the elucidation of the catalyst stability and CIP degradation pathways were performed only for the most active material used in this study, namely copper(II) phosphate. Figures 7A and S7A show the influence of H_2_O_2_ concentration on the efficiency of CIP degradation through the photo-assisted Fenton-like process. According to the results, very low activity is observed in the absence of H_2_O_2_, indicating that copper(II) phosphate cannot be used solely as an efficient photocatalyst, and ROS formed upon catalytic activation of H_2_O_2_ via a Fenton-like reaction are crucial for efficient degradation of the antibiotic. The addition of a very small amount of H_2_O_2_ to the reaction medium resulted in a significant increase in the degradation rate of CIP. The higher the initial concentration of H_2_O_2_, the greater the efficiency of CIP removal (Figs. 7A and S7A). Optimization studies were also carried out by changing the catalyst loading. Only 5% of the initial CIP molecules were found to be degraded by simple photolysis in the presence of H_2_O_2_ and the absence of the catalyst after 60 min of the reaction (Fig. S7B). In this case, the reaction rate was found to be very low (0.000966 min^−1^, Fig. 7B). The addition of only 25 mg of the catalyst to the reaction medium resulted in a significant increase in the CIP degradation rate by a factor of 5.7 (Figs. 7B and S7B). The highest reaction rate was observed for the reaction with the use of 75 mg of the catalyst, indicating that the optimum catalyst loading should be achieved. An additional increase in the catalyst dosage (up to 100 mg) did not result in an increase in the reaction rate, most probably due to the shading effect resulting from the presence of a large amount of catalyst particles. A similar phenomenon has been observed in many previous studies, and it was interpreted as a result of a higher contribution of light scattering by the catalyst particles, which affected the depth of light penetration into the reaction media and reduced the efficiency of the photo-assisted processes. These results clearly show that copper(II) phosphate plays a crucial role in the degradation of CIP via the photo-assisted Fenton-like process.

**Figure 8:**
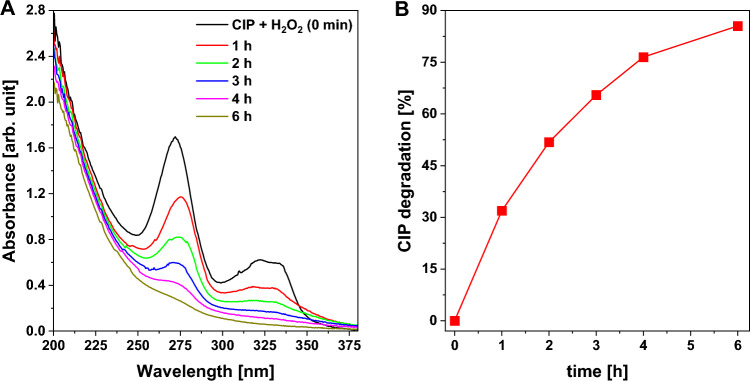
Effects of reaction time on the efficiency of CIP removal via a photo-assisted Fenton-like process in the presence of a Cu_3_(PO_4_)_2_ catalyst: (**A**) UV–vis spectra of post-reaction mixtures collected after a given reaction time, (**B**) graph presenting the efficiency of CIP removal as a function of time. *Reaction conditions:* catalyst (50 mg), H_2_O_2_ (100 μL, 30%), CIP (100 mL, 15 mg/L), room temperature, stirring rate (600 rpm), visible light (λ ≥ 400 nm), without pH adjustment (native pH ~ 6.5).

Optimization studies also included the evaluation of the impact of reaction time on the efficiency of CIP degradation in a photo-assisted Fenton-like reaction. As shown in Fig. 8, the efficiency of CIP degradation could be easily improved by increasing the reaction time. After 6 h of the reaction, most of the antibiotic molecules were degraded, while H_2_O_2_ still remained in the reaction medium. These results clearly show that copper(II) phosphate can efficiently remove CIP even in the presence of a relatively low initial concentration of H_2_O_2_ in the reaction medium. To gain a deeper understanding of the mineralization of CIP during the Fenton-like and photo-assisted Fenton-like processes, TOC analyses were performed. It was found that the concentration of total organic carbon decreased from 7.66 to 6.30 mg/L after 6 h of the Fenton-like process. A significantly higher CIP mineralization efficiency was observed in the photo-assisted Fenton-like process (TOC concentration after 6 h = 5.59 mg/L). These results indicate that CIP may be successfully degraded in the presence of copper(II) phosphate, but high efficiency of antibiotic mineralization would require much longer reaction times.

Since CIP was not fully mineralized, ESI–MS studies were performed to identify the main degradation products. As shown in Fig. S8, in the MS spectrum recorded for the CIP solution, a peak can be observed at m/z = 332 which is characteristic of the pristine form of this antibiotic. The MS spectra recorded for the post-reaction mixtures after increasing the reaction time revealed the formation of other compounds characterized by higher m/z values than those observed for the pristine CIP, that is, m/z = 360 and 362. The formation of these compounds resulted more likely from the substitution of hydroxyl radicals into the structure of CIP (hydroxylation of the antibiotic), which is followed by the destruction of its structure. Indeed, as shown in Fig. S8, the relative intensity of m/z peaks higher than 332 was observed at the beginning of the photo-assisted Fenton-like process and was continuously decreasing over the reaction time. After a longer reaction time, the most intense m/z peaks appeared at much lower m/z values, e.g. 306, 285, 263, 147, confirming successful degradation of the antibiotic. After 6 h of the reaction, the most intense peak occurred at m/z = 263 and was assigned to the CIP molecule in which the piperazine moiety is degraded (see Table 2)^41^. It is important to stress that the intensity of the most intense m/z peak, after such a long reaction time, was ca. 10 times lower than that observed at the beginning of the reaction for CIP (m/z = 332) (Fig. S8). This observation clearly confirms that the majority of CIP molecules were decomposed into small organic molecules characterized by an m/z ratio below 100 (not detected in this study). The proposed structures of the degradation products identified on the basis of ESI–MS data and previous literature reports are shown in Table 2.

**Table 2:** Proposed structures of selected products of CIP degradation through a photo-assisted Fenton-like reaction in the presence of Cu_3_(PO_4_)_2_.

Structural formula	Molecular formula	m/z	References
	C_17_H_19_FN_3_O_3_^+^ [CIP-H]^+^	332	^42^
	C_17_H_18_FN_3_NaO_3_^+^ [CIP-Na]^+^	354	^42^
	C_17_H_15_FN_3_O_5_^+^	360	^43^
	C_17_H_14_FN_3_NaO_5_^+^	382	This work
	C_17_H_17_FN_3_O_5_^+^	362	^44^
	C_15_H_17_FN_3_O_3_^+^	306	^45^
	C_16_H_19_N_3_O_2_^+^	285	^46^
	C_13_H_11_FN_2_O_3_^+^	263	^45^
	C_9_H_11_N_2_^+^	147	^47^

**Figure 9:**
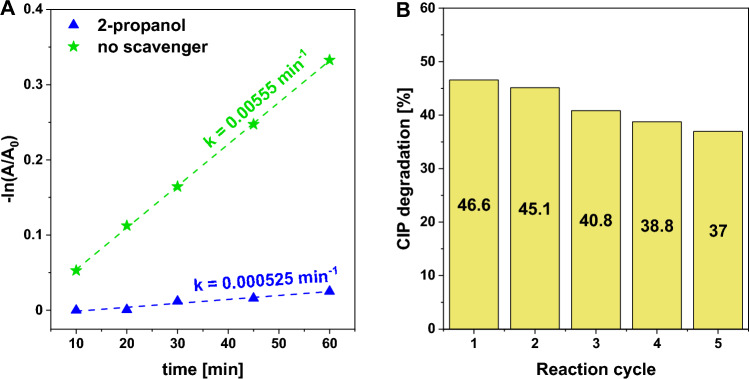
(**A**) Pseudo-first-order plot for the determination of the apparent CIP degradation rate in a photo-assisted Fenton-like process in the presence of 2-propanol as the hydroxyl radical scavenger. (**B**) Efficiency of CIP degradation during five subsequent reaction cycles. *Reaction conditions:* catalyst (Cu_3_(PO_4_)_2_, 50 mg), H_2_O_2_ (100 μL, 30%), CIP (100 mL, 15 mg/L), room temperature, stirring rate (600 rpm), visible light (λ ≥ 400 nm), without pH adjustment (native pH ~ 6.5).

The results obtained from ESI–MS measurements suggested that CIP molecules were more likely to be degraded upon the action of hydroxyl radicals that are usually formed in Fenton-like and photo-assisted Fenton-like reactions. To gain a deeper understanding of the role of ROS in CIP degradation, additional catalytic tests were performed in the presence of 2-propanol as a hydroxyl radical (HO^·^) scavenger. According to the results, the addition of a very small amount of this alcohol to reaction medium almost totally stopped the CIP degradation (Fig. 9A), indicating that hydroxyl radicals were the key active species responsible for highly efficient oxidation of CIP in a photo-assisted Fenton-like process.

The catalyst stability is a very important factor in determining its potential application, so our studies also included reuse tests. As shown in Fig. 9B, the efficiency of CIP removal after each reaction cycle was slightly reduced, but no significant deactivation effect was observed. In general, after five reaction cycles, the efficiency of CIP removal decreased only by ca. 10% (from 47 to 37%; Fig. 9B). To identify the origin of this slightly decreasing activity of copper(II) phosphate, ICP-OES analyses were performed and revealed that approximately 2.3% of the initial Cu^2+^ ions were leached from the catalyst after 6 h of the photo-assisted Fenton-like process (Table S2). This information implies that this slight deactivation effect resulted from the partial leaching of copper species from the catalyst. As no significant leaching of copper species and no major deactivation of the catalyst were observed, it cannot be excluded that the slightly decreasing activity could also be associated with slight losses of the catalyst mass during the reuse procedure. Similar or even more pronounced leaching and deactivation phenomena have usually been reported in the literature for other photo-, Fenton-like and photo-Fenton-like catalysts, namely Fe_2_O_3_^48^, CuO^49,50^, or ZnO^45,51^.

## Conclusions

The results obtained in this study clearly show that copper(II) phosphate is a promising nanomaterial for the catalytic activation of hydrogen peroxide and degradation of ciprofloxacin via Fenton-like and photo-assisted Fenton-like processes that significantly outperform other metal oxides in terms of their catalytic activity. Although Cu_3_(PO_4_)_2_ exhibits no noticeable photocatalytic activity, its activity in a Fenton-like reaction could be significantly enhanced upon exposure of the reaction medium to visible light (λ > 400 nm). Copper(II) phosphate was found to be ca. 7 times more active in CIP degradation via a Fenton-like reaction, and ca. 3.5 more active in a photo-Fenton-like process than the commercial CuO. Ciprofloxacin degradation in the presence of copper(II) phosphate proceeded according to pseudo-first-order kinetics, and the main ROS responsible for the efficient degradation of the antibiotic were hydroxyl radicals. The degradation efficiency of the CIP could be easily improved by increasing the catalyst loading and the H_2_O_2_ dosage. Moreover, the catalyst could be easily reused without any significant deactivation. Thus, the results of our study have shown that copper(II) phosphate is a promising and effective catalyst for the degradation of ciprofloxacin in aqueous media.

### Supplementary Information

Supplementary Information.

## Data Availability

All data generated or analyzed during this study are included in this article.
