# Supplementary material for: Copper(II) phosphate as a promising catalyst for the degradation of ciprofloxacin via photo-assisted Fenton-like process
Source: Sci Rep. 2024 Mar 25;14:7007. doi: 10.1038/s41598-024-57542-9 (PMC10961321; doi:10.1038/s41598-024-57542-9)
Supplement: Supplementary file 1 — Supplementary Information. [file 41598_2024_57542_MOESM1_ESM.pdf]

Supplementary Data

for

Copper(II) phosphate as a promising catalyst for the degradation of ciprofloxacin via photo-assisted Fenton-like process

Mateusz Rozmyślak, Adrian Walkowiak, Marcin Frankowski, Lukasz Wolski*

Faculty of Chemistry, Adam Mickiewicz University, Poznań, ul. Uniwersytetu Poznańskiego 8, 61-614 Poznań, Poland

** corresponding author (wolski.lukasz@amu.edu.pl)*

Table of content:

Extended experimental section.. S2

Fig. S1. (A) UV-Vis spectra of CIP at different concentrations. **(B)** Linear correlation between absorbance and CIP concentration. **(C)** Comparison of UV-Vis spectra: initial CIP and water with varied hydrogen peroxide concentrations. **(D)** UV-Vis spectra of CIP with varied hydrogen peroxide concentrations... S3

Fig. S2. XRD pattern of commercial CuO. S4

Fig. S3. Degradation of CIP in the presence of investigated catalysts in Fenton-like and photo-Fenton-like processes... S5

Fig. S4. XRD patterns of materials used as reference catalysts. S6

Fig. S5. N_2 adsorption–desorption isotherms recorded for materials used as reference catalysts. S7

Fig. S6. Pseudo-first order plot for determination of apparent CIP degradation rate in Fenton-like and photo-Fenton-like processes S8

Fig. S7. (A) The effect of hydrogen peroxide concentration and **(B)** catalyst loading on the efficiency of ciprofloxacin degradation. S9

Fig. S8. ESI-MS spectra of the post-reaction mixtures collected after different time of CIP degradation via the photo-assisted Fenton-like process in the presence of $Cu_3(PO_4)_2$.. S11

Table S1. Comparative study of the results obtained in this work with the previous literature data for the degradation of CIP in the presence of various catalysts……..………..……………………………..S12

Table S2. ICP-OES analysis of the post-reaction mixture after 6 h of CIP degradation via the photo-assisted Fenton-like process in the presence of $Cu_3(PO_4)_2$ catalyst.. S13

Extended experimental section

Synthesis of reference materials

To obtain **$CePO_4$**, 4.3422 g (0.010 mol) of $Ce(NO_3)_3 \cdot 6\ H_2O$ was dissolved in 150 mL of deionized water. Then, while stirring continuously, 1.3206 g (0.010 mol) of $(NH_4)_2HPO_4$ previously dissolved in 50 mL of deionized water was added to the above solution. After one hour of stirring, the resulting solution was placed in a stainless steel Teflon-lined hydrothermal reactor and heated for 24 h at 100°C. After the reactor cooled spontaneously to room temperature, the obtained precipitate was separated by centrifugation and washed twice with deionized water. The resulting material was dried at room temperature for a week and then calcined at 500°C for 4 h. The synthesis of **CeO_2** was analogous to the synthesis of $CePO_4$, but 1.1999 g (0.030 mol) of NaOH was used instead of $(NH_4)_2HPO_4$.

Phosphate-doped iron(III) oxide (denoted as **$P{:}Fe_2O_3$**) was prepared as follows: 0.030 mol of $Fe(NO_3)_3 \cdot 9H_2O$ was dissolved in 150 cm^3 of DI water. Simultaneously, 0.006 mol of $(NH_4)_2HPO_4$ was stirred into 41 cm^3 of deionized water. The resulting solutions were mixed (pH = 1.37), and 9 cm^3 of ammonia solution was introduced into the reaction mixture until the pH increased to approximately 9.00. Next, the solution was transferred to a Teflon-lined stainless steel hydrothermal reactor. The reactor was then firmly closed and introduced into an oven, preheated to 175 °C. Hydrothermal reaction was carried out for 24 h. After that time, the reactor was removed from the furnace, cooled down and opened. The solid material formed was separated by filtration, washed with deionized water (ca. 600 cm^3) and then dried overnight at 60 °C. In the end, the solid sample was calcined for 2 h at 400 °C (temperature ramp: 1.5 °C min^{-1}).

Iron(III) oxide (denoted as **Fe_2O_3**) was prepared as follows: 0.030 mol of $Fe(NO_3)_3 \cdot 9\ H_2O$ was dissolved in 191 cm^3 of DI water. The rest of the synthesis looked identical to the above synthesis of $P{:}Fe_2O_3$ but no $(NH_4)_2HPO_4$ was used during the synthesis.

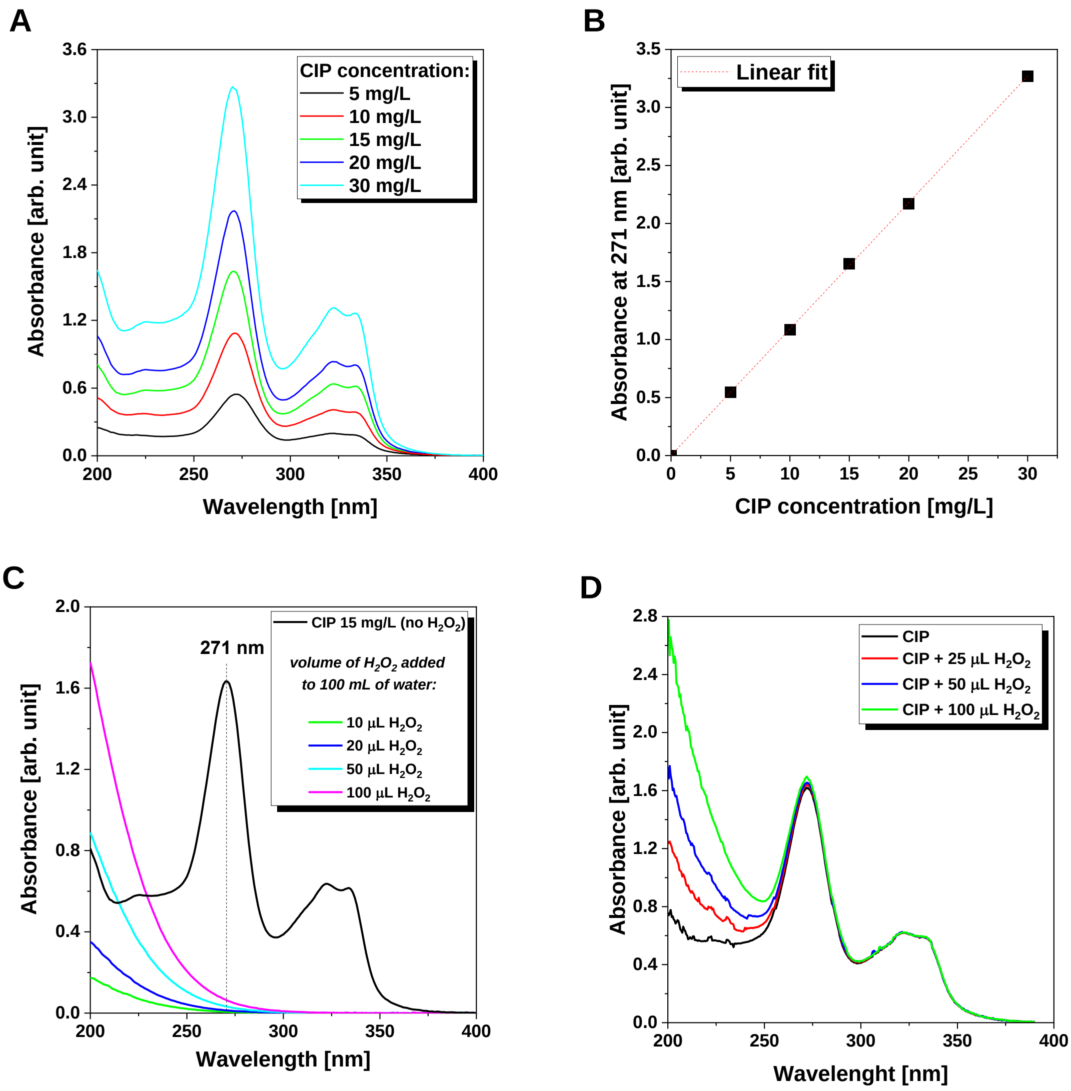

Fig. S1. (A) UV-Vis spectra of CIP at different concentrations. **(B)** Linear correlation between absorbance and CIP concentration. **(C)** Comparison of UV-Vis spectra: initial CIP and water with varied hydrogen peroxide concentrations. **(D)** UV-Vis spectra of CIP with varied hydrogen peroxide concentrations.

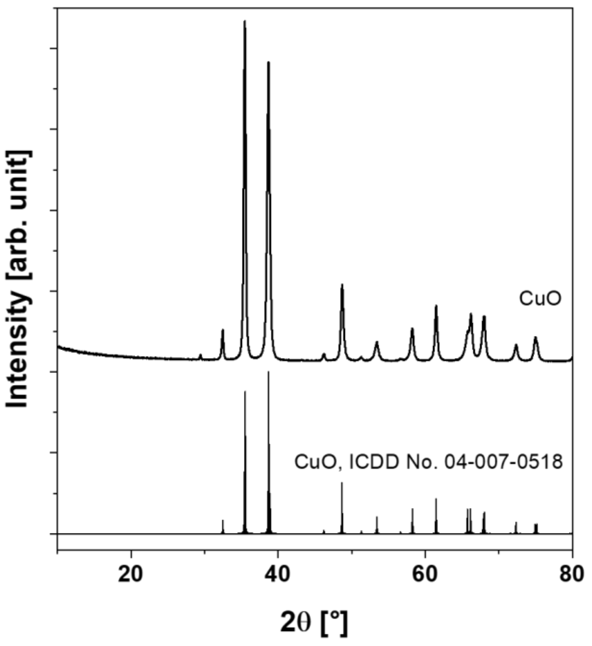

Fig. S2. XRD pattern of commercial CuO.

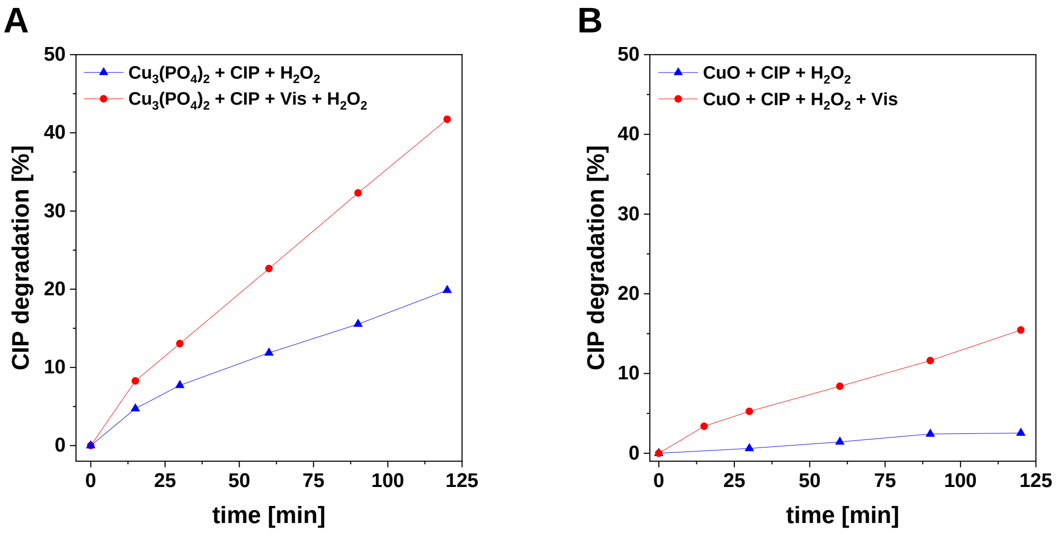

Fig. S3. Degradation of CIP in the presence of investigated catalysts in Fenton-like and photo-Fenton-like processes. *Reaction conditions:* catalyst (25 mg), H_2O_2 (50 μL, 30%), CIP (100 mL, 15 mg/L), room temperature, stirring (600 rpm), visible light (λ ≥ 400 nm), without pH adjustment (native pH ~ 6.5)

.

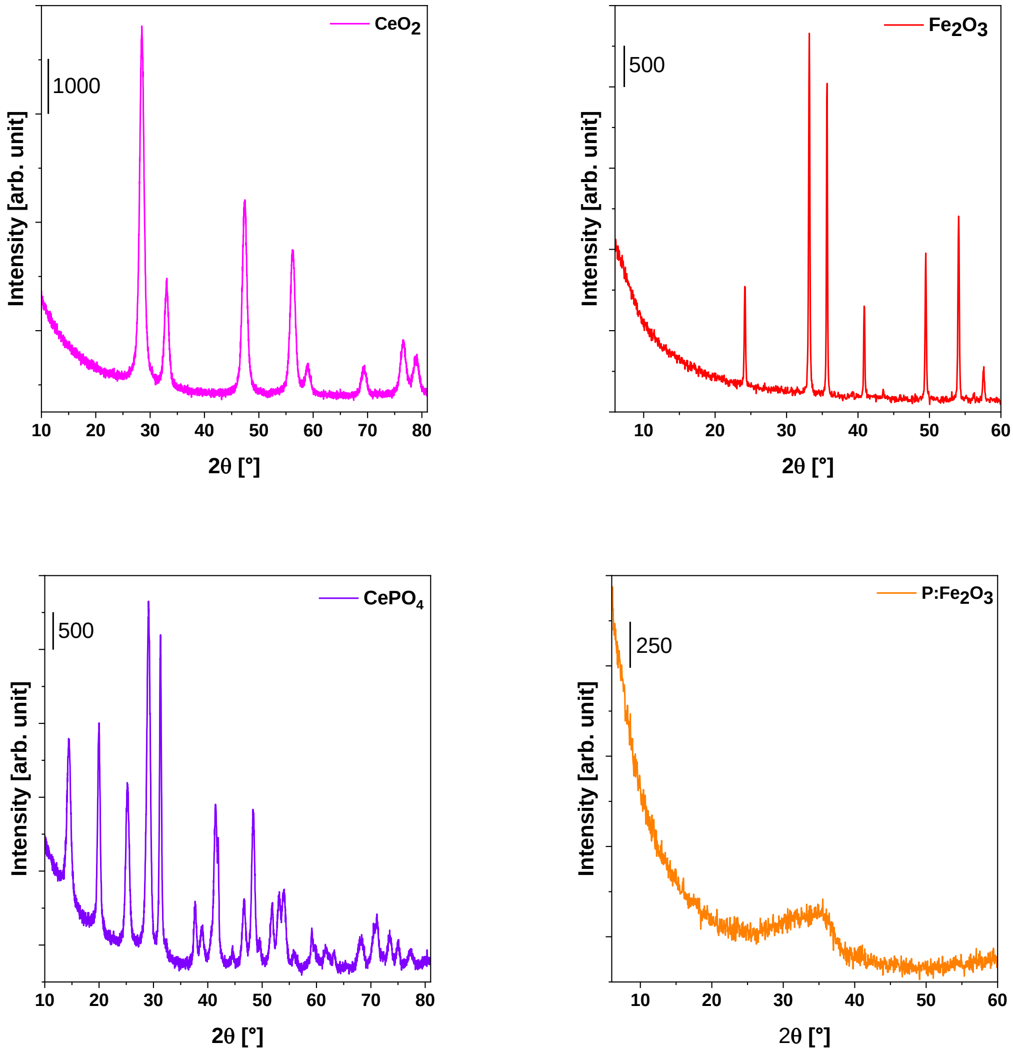

Fig. S4. XRD patterns of materials used as the reference catalysts.

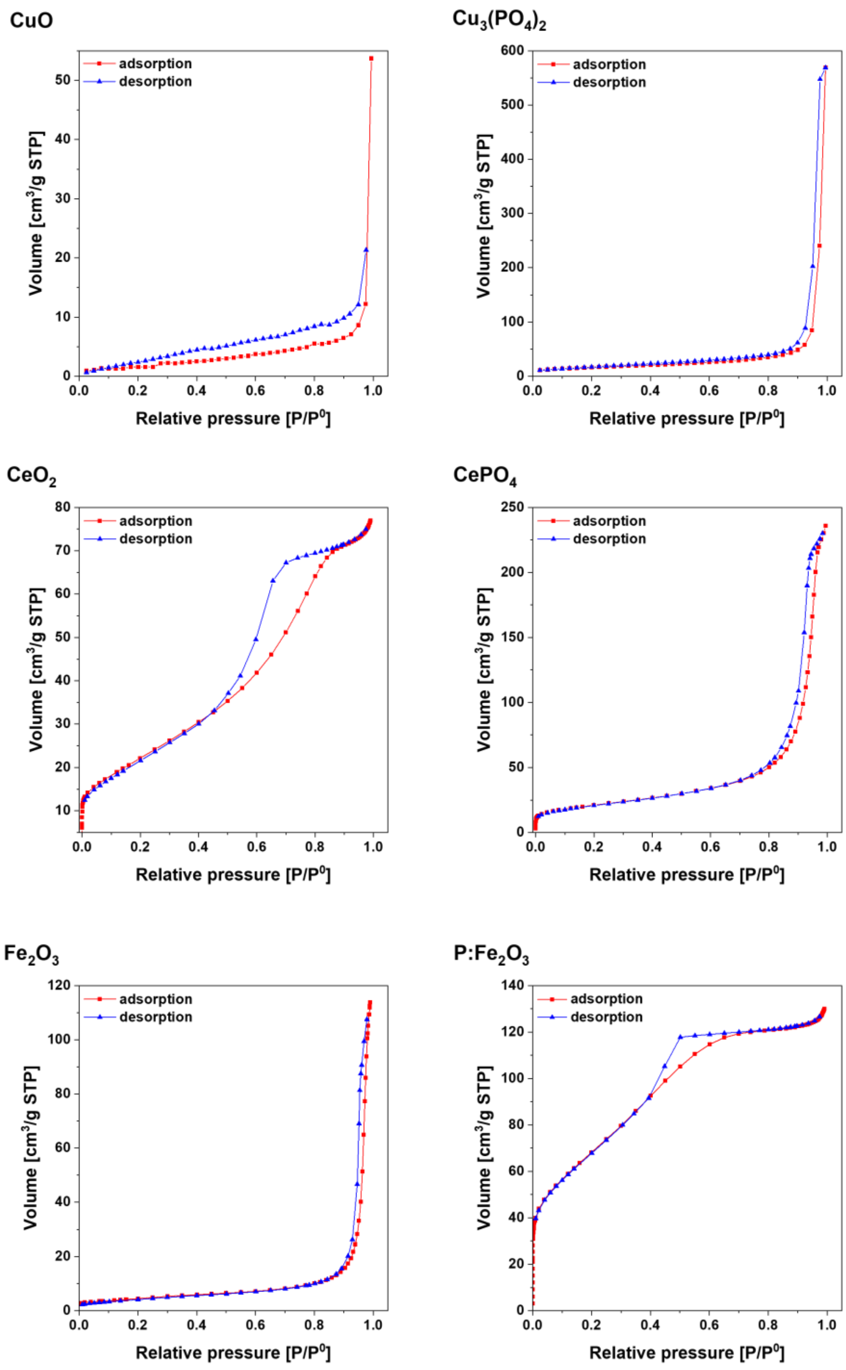

Fig. S5. N_2 adsorption–desorption isotherms recorded for materials used as the reference catalysts.

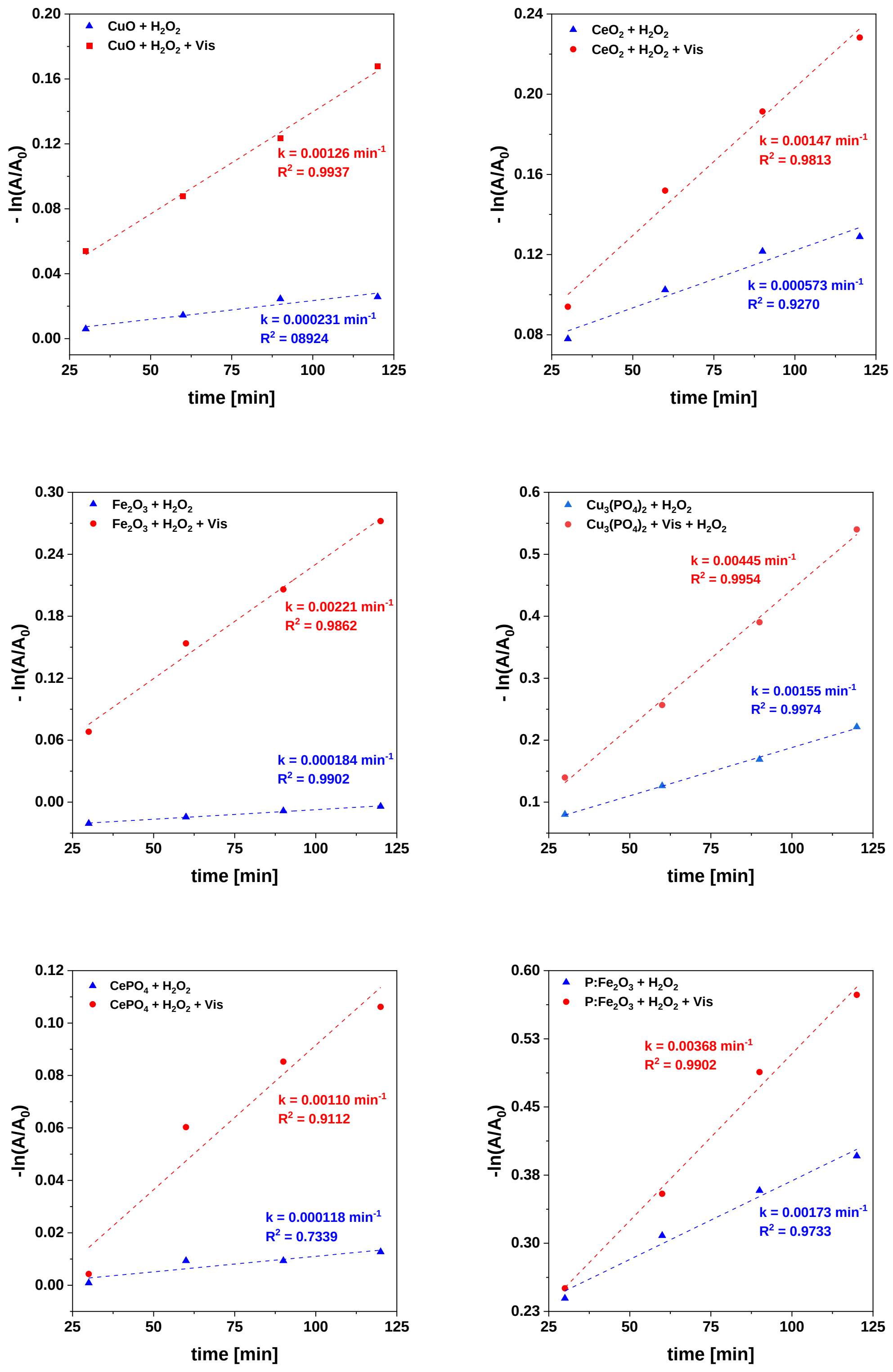

Fig. S6. Pseudo-first order plot for determination of apparent CIP degradation rate in Fenton-like and photo-Fenton-like processes. Reaction conditions: catalyst (25 mg), H_2O_2 (50 µL, 30%), CIP (100 mL, 15 mg/L), room temperature, stirring rate (600 rpm), visible light (λ ≥ 400 nm), without pH adjustment (native pH ~ 6.5).

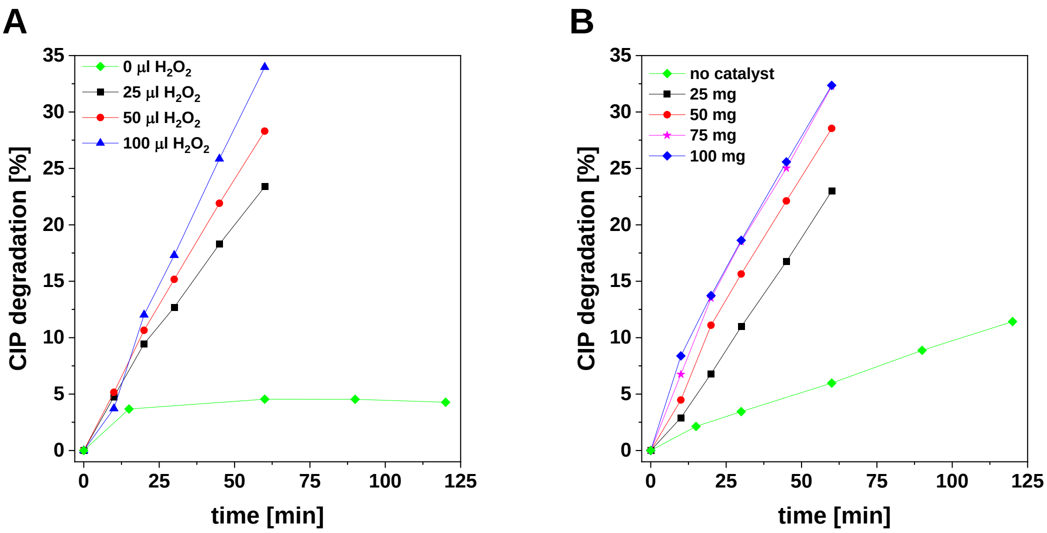

Fig. S7. (A) The effect of hydrogen peroxide concentration and **(B)** catalyst loading on the efficiency of ciprofloxacin degradation in the presence of $Cu_3(PO_4)_2$ catalyst. *Reaction conditions:* catalyst (25 mg or other, if indicated), H_2O_2 (50 µL or other, if indicated, 30%), CIP (100 mL, 15 mg/L), room temperature, stirring rate (600 rpm), visible light ($\lambda \geq 400$ nm), without pH adjustment (native pH ~ 6.5).

CIP

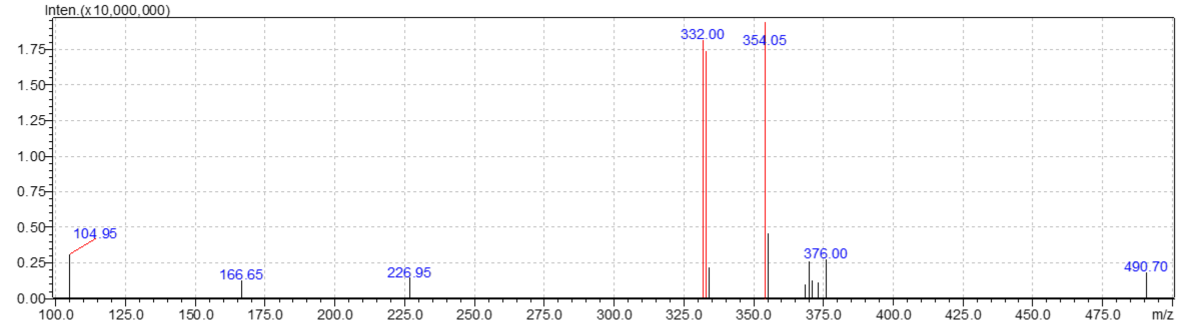

CIP + H_2O_2 + vis + cat_0.5 h

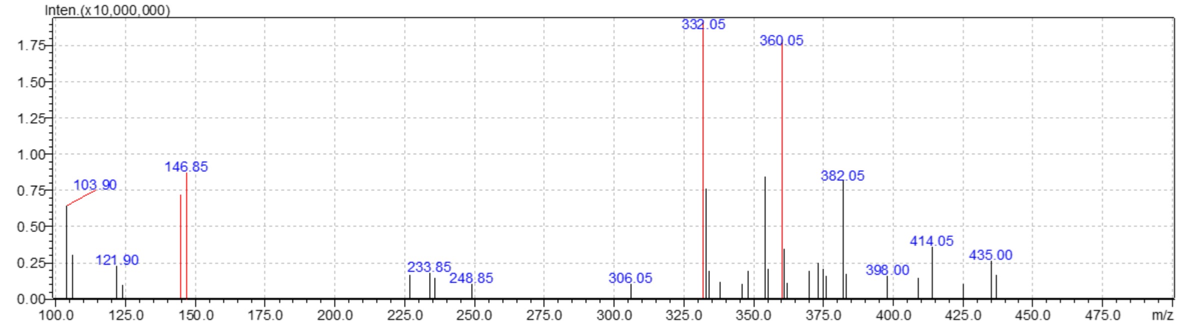

CIP + H_2O_2 + vis + cat_1 h

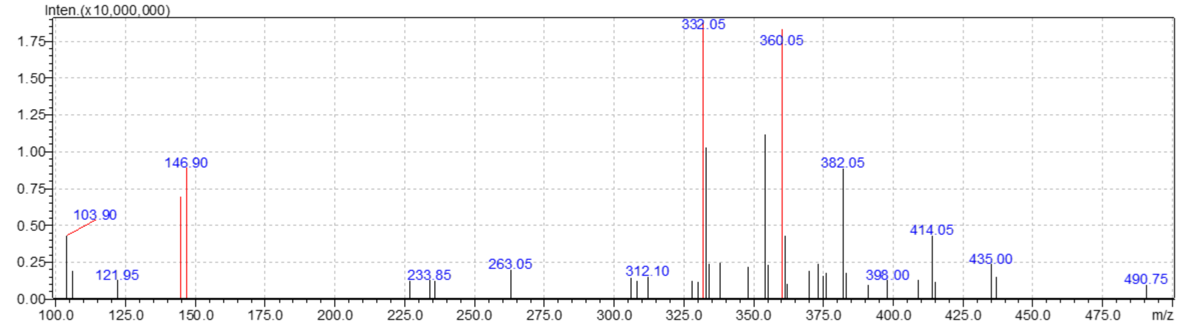

CIP + H_2O_2 + vis + cat_2 h

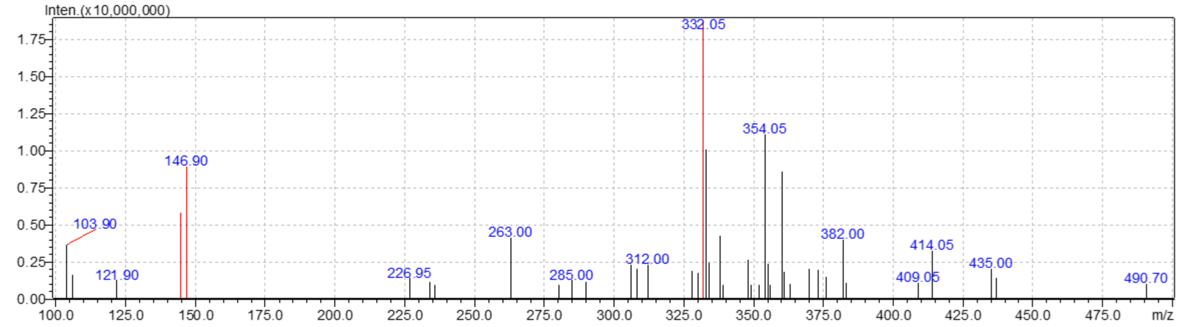

CIP + H_2O_2 + vis + cat_4 h

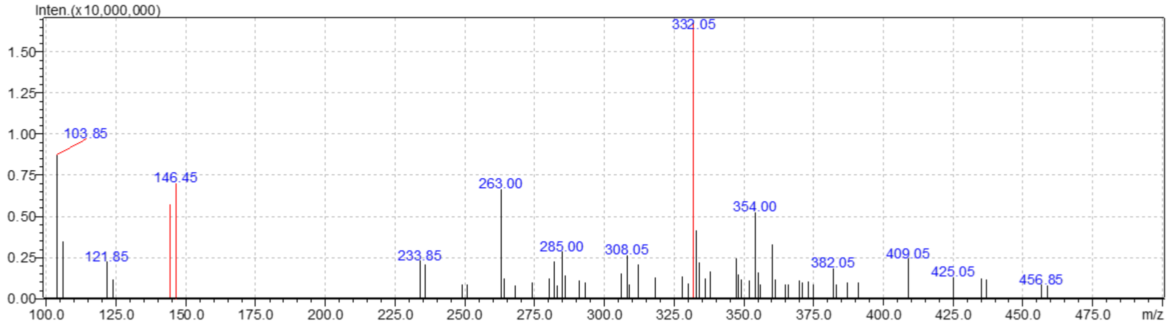

CIP + H_2O_2 + vis + cat_6 h

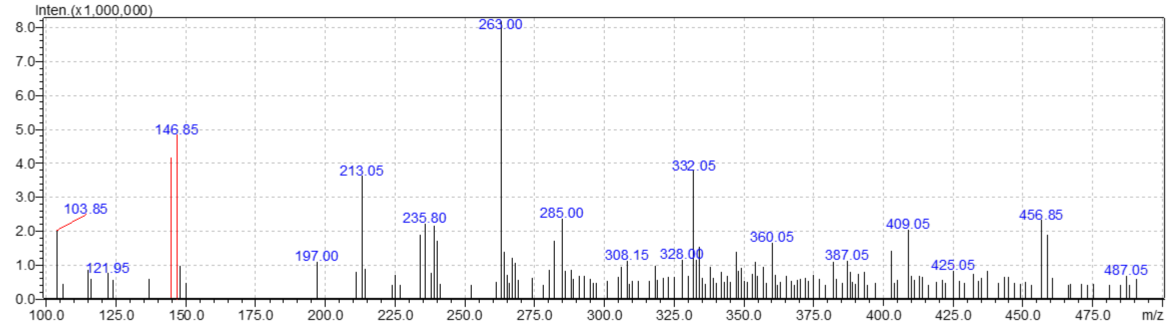

Fig. S8. ESI-MS spectra of the post-reaction mixtures collected after different time of CIP degradation via the photo-assisted Fenton-like process in the presence of $Cu_3(PO_4)_2$ catalyst. *Reaction conditions:* catalyst (50 mg), H_2O_2 (100 µL, 30%), CIP (100 mL, 15 mg/L), room temperature, stirring rate (600 rpm), visible light (λ ≥ 400 nm), no pH adjustment (native pH ~ 6.5).

Table S1. Comparative study of the results obtained in this work with the previous literature data for the degradation of CIP in the presence of various catalysts.

Catalyst	Catalyst loading [g/L]	CIP concentration [mg/L]	H_2O_2 dosage [mmol/L]	Reaction time [h]	Degradation efficiency [%]	Reaction type	pH	Ref.
Copper(II) phosphate	0.5	15	12.8	6	85.5	Photo-Fenton-like, visible light	~ 6.5	This work
Corncob Biochar-Based Magnetic Iron–Copper Bimetallic Nanomaterial	0.6	10	10	6	93.6	Fenton-like process	6.4	[S1]
MoVBiO	0.1	16.6	5	5	10	Fenton-like process	8	[S2]
MoZnO					10			
MoCoO					20			
MoMnO					20			
MoFeO					78			
Fe_2O_3/MoO_3					21			
HNO_3 modified-biochar	0.4	10	1	24	93	Fenton-like process	7	[S3]
Fc/NH_2/SBA-15	0.375	15	8	1	64	Photo-Fenton-like, visible light	~ 3	[S4]
Fe_3O_4-activated carbon composite (AC4/F)	1.5	-	10	2	~100	Photo-Fenton-like, UV light	3	[S5]
$LaFeO_3$/Diatomite	0.3	20	291	2.5	99	Photo-Fenton-like, visible light	4	[S6]
g-C_3N_4/Fe_3O_4/MIL-100(Fe) ternary heterojunction	0.670	200	78	2.5	95.3	Photo-Fenton-like, visible light	3	[S7]

[S1] Liu, H. *et al.* Adsorption and Fenton-like Degradation of Ciprofloxacin Using Corncob Biochar-Based Magnetic Iron–Copper Bimetallic Nanomaterial in Aqueous Solutions. *Nanomaterials* **12**, 4 (2022).

[S2] Yin, S. et al. Degradation of ciprofloxacin with hydrogen peroxide catalyzed by ironmolybdate-based zeolitic octahedral metal oxide. *Appl. Catal. A Gen.* **626**, 118375 (2021).

[S3] Luo, K. et al. Unveiling the mechanism of biochar-activated hydrogen peroxide on the degradation of ciprofloxacin. *Chem. Eng. J.* **374**, 520–530 (2019).

[S4] Walkowiak, A., Wolski, L. & Ziolek, M. The influence of ferrocene anchoring method on the reactivity and stability of SBA-15-based catalysts in the degradation of ciprofloxacin via photo-Fenton process. *RSC Adv*. **13**, 8360–8373 (2023).

[S5] Fortunato, A. B. et al. Buriti biomass as catalysts based on activated carbon/Fe_3O_4 for ciprofloxacin removal by heterogeneous photo-Fenton process. *J. Water Process Eng*. **50**, 1–9 (2022).

[S6] Liu, Y. et al. Heterogeneous Photo-Fenton Catalytic Oxidation of Ciprofloxacin Using $LaFeO_3$/Diatomite Composite Photocatalysts under Visible Light. *ChemistrySelect* **5**, 14792–14799 (2020).

[S7] He, W. et al. Magnetic recyclable g-C_3N_4/Fe_3O_4@MIL-100(Fe) ternary catalyst for photo-Fenton degradation of ciprofloxacin. *J. Environ. Chem. Eng.* **10**, 108698 (2022).

Table S2. ICP-OES analysis of the post-reaction mixture after 6 h of CIP degradation via the photo-assisted Fenton-like process in the presence of $Cu_3(PO_4)_2$ catalyst.

Concentration of Cu^{2+} in post-reaction media [mg/L]	% of leached Cu species	Concentration of P in post-reaction media [mg/L]	% of leached P species
5.83	2.3	4.77	5.8

Reaction conditions: catalyst (50 mg), H_2O_2 (100 μL, 30%), CIP (100 mL, 15 mg/L), room temperature, stirring rate (600 rpm), visible light ($\lambda \geq$ 400nm), pH (~ 6.5).
